# Investigation of the Mechanical Behavior of Synthesized Al6061/TiO_2_ Microcomposites Using an Innovative Stir Casting Method

**DOI:** 10.3390/nano12101646

**Published:** 2022-05-12

**Authors:** A. H. Badran, Turki Alamro, Rabeea W. Bazuhair, Ahmed Ali Gad El-Mawla, S. Z. El-Adben, Ahmed Fouly

**Affiliations:** 1 Department of Production Engineering and Mechanical Design, Faculty of Engineering, Minia University, Minya 61111, Egypt; ahmed.badran@mu.edu.eg (A.H.B.); ahmed.ali@minia.edu.eg (A.A.G.E.-M.); samyzein1965@yahoo.com (S.Z.E.-A.); 2 Department of Mechanical Engineering, College of Engineering and Islamic Architecture, Umm Al Qura University, Mecca 24382, Saudi Arabia; tsamro@uqu.edu.sa; 3 Department of Civil Engineering, College of Engineering and Islamic Architecture, Umm Al Qura University, Mecca 24382, Saudi Arabia; rwbazuhair@uqu.edu.sa; 4 Mechanical Engineering Department, King Saud University, Riyadh 11421, Saudi Arabia

**Keywords:** metal matrix composites, Al6061, titanium dioxide (TiO_2_), stir casting, mechanical properties

## Abstract

Aluminum composites are preferred in many kinds of applications such as aviation, space, automotive, and marine, owing to their outstanding properties, high strength, and corrosion resistance. The main objective of the current study is to evaluate the mechanical properties of aluminum alloy 6061/titanium dioxide (micro-TiO_2_) microcomposite synthesized using the stir casting method. The effects of changes in stir casting parameters, such as stirring speed and tiring durations, were studied. Al6061 matrix was reinforced with micro-TiO_2_ particles with weight fractions of 1, 2, 3, 4, and 5 wt.%. Microstructural and chemical analyses were conducted to explore microstructural transformation resulting from the presence of the TiO_2_ microparticles. The mechanical characteristics were evaluated, and the results showed a considerable enhancement in the mechanical strength and hardness resulting from the incorporation of micro-TiO_2_ into Al606. The additions of 2 wt.% and 5 wt.% of micro-TiO_2_ recorded the highest ultimate tensile strength and hardness, respectively.

## 1. Introduction

Al6061, one of the 6xxx series (Al/Mg/Si), is higher in corrosion resistance than other alloys belonging to the 2xxx and 7xxx series. Consequently, Al6061 has gained attention in the industrial sector for its uses in marine and aerospace applications [1]. Furthermore, Al6061 has good formability and heat treatability, both of which qualify it to be utilized in different structural applications [2]. Despite having high corrosion resistance, Al6061 has poor mechanical and wear characteristics compared with other aluminum series, such as the 7xxx (Al/Zn) and 2xxx (Al/Cu) series [3,4]. Therefore, researchers have sought to develop the mechanical and wear properties of Al6061 by incorporating ceramic particles as a reinforcement material. Prakash et al. [5] reinforced Al6061 with rock dust particles using the stir casting process. Tribological tests were conducted on samples with different loading fractions and sizes of rock dust to investigate the optimum states for the least wear rates. The results showed an improvement in the wear characteristics resulting from the addition of the rock dust particles with different loading fractions. Ramesh et al. [6] sought to enhance Al6061 wear resistance in terms of the wear coefficient by adding TiO_2_ microparticles. They found that samples were 8% micro-TiO_2_ recorded the lowest wear coefficient with different sliding distances and applied loads.

In metal matrix composites (MMCs), researchers have utilized different ceramic fillers. TiO_2_ is one of the most popular ceramic fillers, chemically inert [6], with superior corrosion resistance and high hardness and modulus [6]. An improvement in compressive strength was acquired for Al7075 by increasing the TiO_2_ content with a fixed amount of fly ash [7]. An enhancement in wear characteristics of Al6061 from adding TiO_2_ was obtained by Ramesh et al. [6]. Shin et al. [8] recorded an improvement in the mechanical properties of Al2024 with the incorporation of TiO_2_ nanoparticles by using the powder metallurgy technique. Wettability, porosity, clustering of particles, and good distribution are important issues concerning MMCs that must be considered. It is impossible to perfect the wettability between metal and ceramic surfaces, as they are two different materials, but some considerable improvements were made in this approach. Ramnath et al. [9] proved that adding preheated Al_2_O_3_ and B_4_C to aluminum alloy (LM 25) at 400 °C can enhance the wettability between the particle and the matrix. Adding 1 wt.% Mg particles to a hybrid composite of Al6061 with SiC and fly ash improved wettability and showed a good mechanical properties response (hardness and UTS) [10]. Another attempt to improve the interface state between the phases was achieved by [11], who replaced the poor wettable metal with a ceramic interface having a metal-to-metal interface by means of an electroless coating of the reinforcement powder by Cu. The technique was able to be used to produce a composite with enhanced hardness and wear characteristics.

Due to its simplicity, low cost, flexibility, and availability for mass production, compared with other methods, the stir casting process is a promising technique for utilization in the producing of MMCs [12]. Bharath et al. [13] prepared Al6061 MMCs reinforced by Al_2_O_3_ microparticles using a stir casting process, adopting three steps of mixing coupled with the preheating of the reinforcing particles. The amount of Al_2_O_3_ particulates added to the composites varied from 6 wt.% to 12 wt.%. The distribution of Al_2_O_3_ particulates was investigated utilizing optical micrographs. The composite microstructure confined the primary α-Al dendrites and eutectic silicon, whereas Al_2_O_3_ particulates were dispersed at inter-dendritic areas and in the eutectic silicon. The composite production technique parameters were able to affect the characteristics of the produced composite [14]. Prabu et al. [15] investigated the influence of stir casting variables, such as stirring time and speed, on the reinforcement distribution. They found that increasing the stirring speed and time led to better distribution and uniform hardness, especially at 600 rpm for 10 min of stirring. Hashim et al. [16,17] investigated the effect of changing the stir casting parameters on the characteristics of composites. They found that the composite’s microstructure and hardness were deeply affected by the variation in stirring speed and time. Naher et al. [18] investigated the distribution of the reinforcement particles inside the matrix with the change in stir casting parameters. The microstructure results showed that a low stirring speed and time led to reinforcement particles agglomeration.

As illustrated in the literature survey, incorporating ceramic fillers into metals can considerably enhance their properties. Furthermore, the production technique parameters could also affect the produced metal matrix composite. Consequently, the main objective of the current investigation is to evaluate the mechanical characteristics of Al6061 after adding particles of micro-TiO_2_ utilizing the stir casting technique. Al6061 composites were fabricated with different loading fractions of micro-TiO_2_, 1, 2, 3, 4, and 5 wt.%, and compared with pure Al6061, 0 wt.%. Different stirring speeds and times were applied during the production process to investigate their effects on the properties of Al6061 composites. Besides the structural and morphological characteristics of the Al6061 composite, the chemical composition was analyzed.

## 2. Experimental Procedure

**Figure 1:**
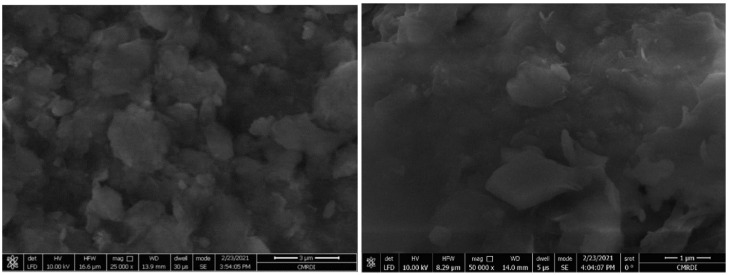
SEM micrographs of TiO_2_.

The Al6061 alloy, considered the matrix material, was supplied from Helwan Company of Non-Ferrous Industries, Cairo, Egypt. The chemical composition of the matrix material consisted of Al, Si, Fe, Cu, Mn, Mg, Zn, Cr, and Ti with weight fractions of 97.58%, 0.71%, 0.11%, 0.22%, 0.0087%, 1.02%, 0.12%, 0.13%, and 0.1%, respectively. The micro-titanium dioxide was supplied from Morgan Specialty Chemicals, El-Obour City, Kaliobeya, Egypt. The morphology of the titanium dioxide particles was analyzed using a scanning electron microscope (SEM) that revealed the particle size, as shown in Figure 1.

**Figure 2:**
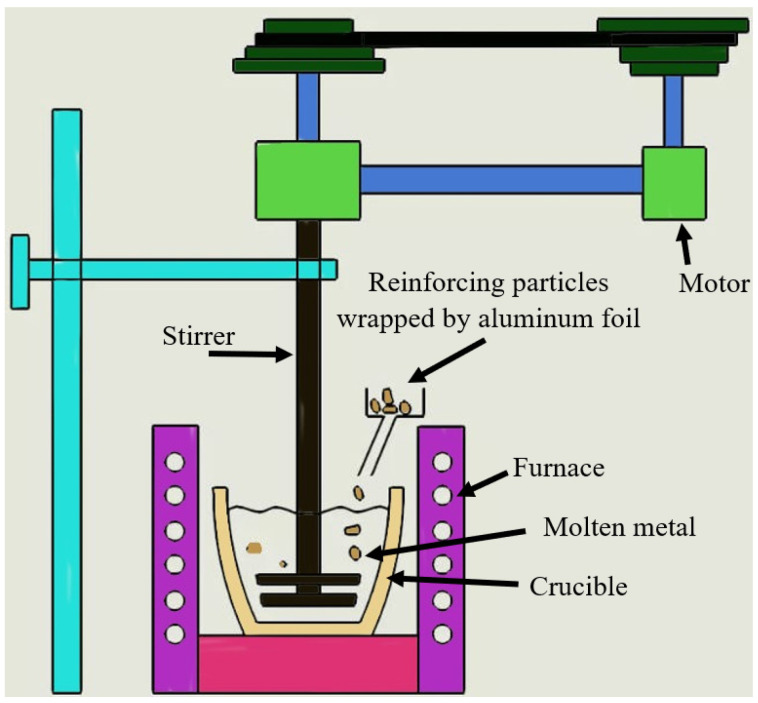
Stir casting machine.

In the present investigation, Al6061 alloy was reinforced with different weight fractions of TiO_2_ microparticles using the stir casting method. The Al6061 matrix metal was cut into small pieces and melted in a crucible furnace at 750 °C. An adequate heating time was supplied to reach the superheat condition of the Al6061 matrix material to ensure a full liquid state and avoid an unmolten or semi-solid state. The stir casting technique was utilized to achieve the optimum distribution of the TiO_2_ microparticles into the Al6061 base material, as shown in Figure 2. Before adding the micro-TiO_2_ particles to the Al6061, the micro-TiO_2_ was heated. This is considered to be a necessary step for the disposing of the adsorbed gases from the particle surface and the avoiding of a high drop in temperature related to the introduction of the particulates [13]. Then, during the stirring process, the TiO_2_ microparticles were added. The Al6061/TiO_2_ microcomposite samples were fabricated with micro-TiO_2_ weight fractions of 0, 1, 2, 3, 4, and 5 wt.%. The stirrer was set at one-third of the height from the bottom of the crucible [19]. The microcomposite was poured into a preheated stainless-steel permanent mold at 300 °C. Stirring speed and time were considered to be the most important parameters of the stir casting process. The speeds and times of stirring were changeable in order to investigate the optimum parameters that allow for the best mechanical properties. Four stirring times were applied (5, 10, 15, and 20 min) with three stirring speeds (550, 1100, and 1300 rpm). To save time, stir casting parameters were applied for 1 and 2 wt.% only, and the mechanical properties were evaluated, including the ultimate tensile strength (UTS).

The uniformity of the reinforcement distribution through the matrix, grain size, structure, and porosity greatly affect the produced composites. Consequently, a microstructural analysis using an Olympus BX51 light microscope was performed. Images were recorded by a digital camera (Olympus DP 73) attached to the microscope using Cell Sense Imaging software (Olympus). Before the examination, the samples were prepared for microscopic check by grinding using different grades of emery paper, polished with diamond paste to obtain a mirror-like surface, then passed through an etchant consisting of 0.5 mL HF in 100 mL water for 10 s [20].

To evaluate the mechanical properties of the produced microcomposites, a uniaxial tension test was conducted using a servo-controlled universal testing machine—namely, the United Smart Universal Hydraulic Floor Model (SHFM) tension and compression testing machine with computer control and a capacity of 300 KN. The tension test was performed at room temperature and a strain rate of 5 mm/s to determine the maximum tensile strength for the composites. The tested samples were prepared according to the ASTM E8-04 standard. The change in the ultimate tensile strength was recorded in order to study the effect of the micro-TiO_2_ reinforcement particles on the strength of the Al6061 composites. Mechanical properties were also able to be evaluated by measuring the hardness of the samples. Macro-hardness tests were performed using a Vickers hardness testing machine that used a diamond indenter with a load of 980 N for 30 s on cylindrical specimens. A right pyramid indenter was used, with a square base with an angle of 136° between the opposite faces. The samples were polished before the hardness test. The test was carried out at an atmospheric temperature of 30 °C, and the hardness measurement was taken, representing the average of three separate measurements. The indenter impressions were measured by an optical microscope attached to the hardness machine with magnification up to 20 times.

## 3. Results and Discussion

### 3.1. Optimization of Stir Casting Parameters

**Figure 3:**
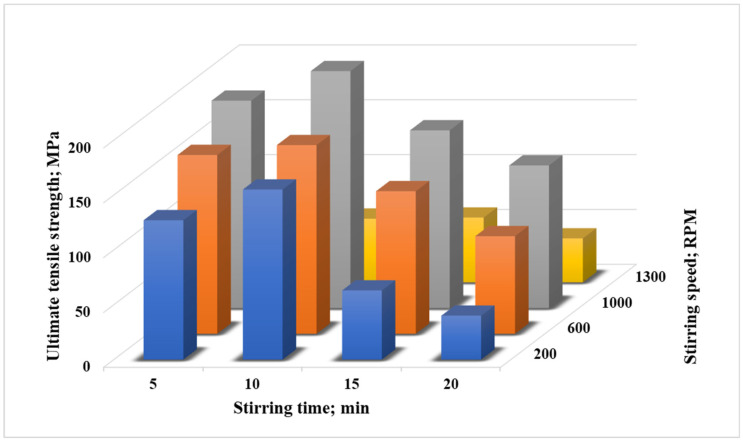
Ultimate tensile strength values for Al6061/TiO_2_ (1 wt.%) stirred at different speeds and duration times.

The main challenge during the stir casting technique is identifying the optimum stirring parameters such as the stirring speed and stirring durations. Therefore, the stir casting parameters were evaluated with Al6061 samples containing 1 and 2 wt.% of TiO_2_. A combination of stir casting parameters were applied. The resulting microcomposite samples were tested to find the optimum conditions for a strengthened microcomposite in terms of ultimate tensile strength (UTS). The ultimate tensile strength values of Al6061/TiO_2_ (1 wt.%) microcomposite at different stirring speeds and stirring durations are shown in Figure 3. Figure 3 illustrates that, at different stirring durations, the speed of 1000 rpm recorded the highest ultimate tensile strength (UTS), and the maximum UTS was observed at a stirring duration of 10 min, approximately 215 MPa. At the low stirring time, 5 min, less uniform scatter of TiO_2_ occurred, and at a long stirring time, 15 and 20 min, the TiO_2_ microparticles may be aggregated close to the wall of the crucible because of the centrifugal impact of the vortex motion, causing the particles to leave the matrix core with no reinforcement particles [19]. Erosion of the stirrer blade material may also occur due to a very long stirring time, leading to the introduction of eroded parts of the stirrer into the composite as a different phase in the latest stages. Furthermore, at long stirring times, 15 and 20 min, a potential peeling on the surface of the steel stirrer during the stirring process may have occurred, encouraging the migration of ferrous ions from the stirrer into the molten metal and directing some constituents of the stirrer material to be presented in the molten metal, creating an odd irregular phase that works as a defect and decrease the whole of the results from the testing [21,22]. Furthermore, the drastic deterioration that is recorded at a stirring speed of 1300 rpm could be attributed to the high speed, which may create a strong vortex, resulting in turbulence and the suction of air bubbles. This is an unpleasant situation and leads to gas entrapment [23].

**Figure 4:**
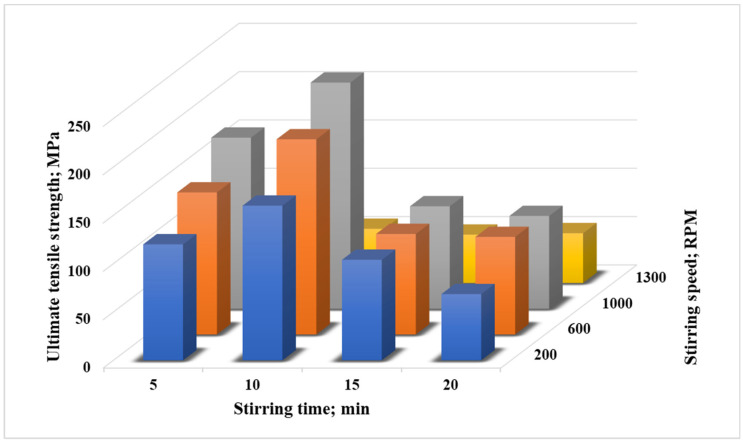
Ultimate tensile strength values for Al6061/TiO_2_ (2 wt.%) stirred at different speeds and duration times.

The same experiments were repeated for the Al6061 composite containing 2 wt.% of micro-TiO_2_. The results are illustrated in Figure 4. Figure 4 illustrates the ultimate tensile strength values of Al6061/TiO_2_ (2 wt.%) at different stirring speeds and stirring durations. The results were similar to those observed for Al6061/TiO_2_ (1 wt.%). Stirring up to 1000 rpm provided an increasing path in the tensile strength of the reinforced composites, while the stirring at 1300 rpm recorded a lower strength value. It was also observed that increasing the stirring periods resulted in a corresponding improvement in tensile strength response up to stirring for 10 min, but beyond this point, i.e., stirring for 15 and 20 min, the tensile outcomes followed a decreasing trend, indicating that longer stirring durations lead to excessively sucking air entrapment, resulting in porosity in the castings [24]. To conclude, the results illustrated that changing the stir casting parameters—stirring speed and time—was able to affect the mechanical properties of Al6061/TiO_2_ (1 and 2 wt.%). The optimum stirring time was 10 min at a speed of 1000 rpm. Consequently, these parameters were fixed throughout the current study.

### 3.2. Effect of TiO_2_ Loading Fraction on the Al6061 Composite Properties

**Figure 5:**
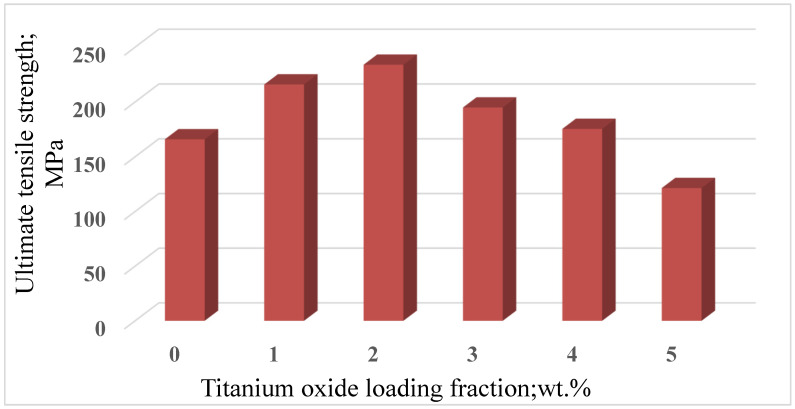
Ultimate tensile strength of Al6061/TiO_2_ with different loading fractions of TiO_2_.

To investigate the effect of the micro-TiO_2_ loading fraction values on the mechanical properties of Al6061 composites, samples with different loading fractions, 1.5 wt.%, were fabricated using the selected stir casting parameters. Figure 5 illustrates the ultimate tensile strength for samples with different loading fractions of TiO_2_. It is obvious that Al6061/TiO_2_ (2 wt.%) showed the highest UTS of all of the composites. The UTS of Al6061/TiO_2_ (2 wt.%) reached 234 MPa with an enhancement of 21%, compared to Al6061, at 165 MPa. These results could be attributed to the presence of the porosity developed during the stirring process and the increasing loading fraction of the reinforcement. More clarification is given in the subsequent sections of the current study, which discuss porosity testing and microstructural investigation.

The enhancement in the strength for all composites, compared with pure Al6061, could be attributed to various strengthening mechanisms, such as dislocation strengthening, hall–pitch strengthening, and strain gradient strengthening, which are associated with the Al6061/TiO_2_ composite. The presence of a rigid phase, TiO_2_, as a reinforcement inside the soft Al6061 matrix, represents a backstop to the easy plastic deformation of the soft phase as the loading increases. With a good reinforcement distribution, the effectiveness of the load transfer from the matrix to reinforcement is achieved, leading to the material exhibiting more strength [25]. Furthermore, the difference in thermal conductivity between the matrix material and reinforcement leads to the uneven cooling rates of the two phases during solidification, fields of residual stress, and thermal-induced geometrically necessary dislocations surrounding the particles, restricting dislocation movement and leading to the need for more loading to be required [26]. In addition, during solidification, the inclusions in the molten solution work as nucleation sites that serve as centers of crystallization [27], so it may be considered that the massive numbers of incorporated particles are potential sites for nucleation. Therefore, these particles impede the growth of the grains around them, forming smaller refined grains with a higher number of grain boundaries, which create a retarding effect in terms of dislocation movement; hence, strength is improved. Additionally, using a preheated mold makes the solidification process of composites slower, leading to more potentiality for particle segregation in grain boundaries and thus retarding grain growth and refining grains [19]. Eventually, the enhancement could occur because of the massive surface area produced by the microparticles in comparison to the macroparticles themselves. This large surface area means more contact area between the matrix and reinforcement, constituting more area and potentiality for impeding dislocations, which strengthen, in turn, the composite.

**Figure 6:**
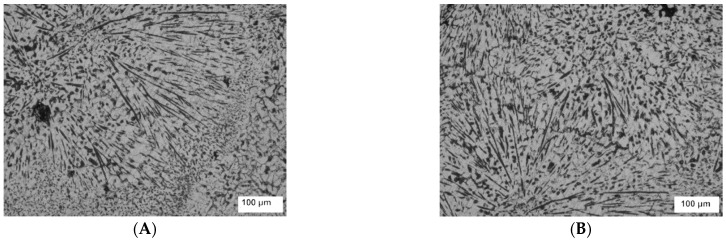
(**A**) Microstructure of Al6061 stirred for 15 min; (**B**) microstructure of Al6061 stirred for 20 min.

**Figure 7:**
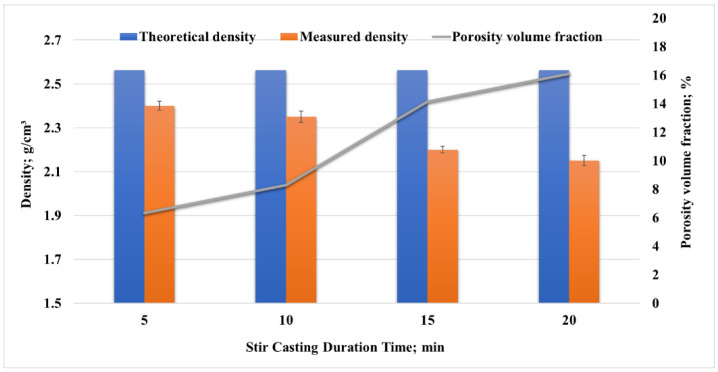
Theoretical and experimental densities of Al6061/TiO_2_ (1 wt.%), together with their porosity volume fractions for different stir casting duration times.

The microstructures of the unreinforced aluminum alloy 6061, which was stirred for long durations of 15 and 20 min at 1300, are shown in Figure 6A,B. A high amount of porosity (the darker areas) was introduced due to the higher speed used for stirring and the long stirring periods that allowed a larger amount of air to be introduced through the melting process. To ensure the existence of the porosity, the composites’ theoretical density was calculated based on the weight fractions and densities of the Al6061 and TiO_2_. Then, the densities of the samples were measured experimentally according to Archimedes’ approach. The porosity volume fraction was estimated based on the theoretical and experimental densities. The porosity percentage of the samples reached 14% and 17% for stirring durations of 15 and 20 min, respectively, as shown in Figure 7. As mentioned previously, a long stirring time can lead to peeling on the surface of the steel stirrer, which may occur during the stirring process, encouraging the migration of ferrous ions from the stirrer into the molten metal.

**Figure 8:**
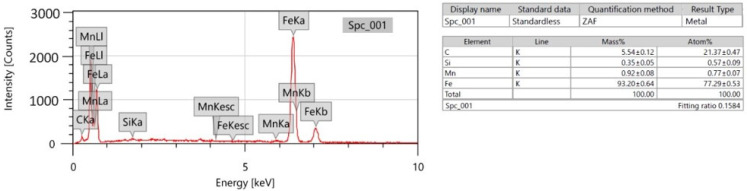
EDX of the stirrer material.

**Figure 9:**
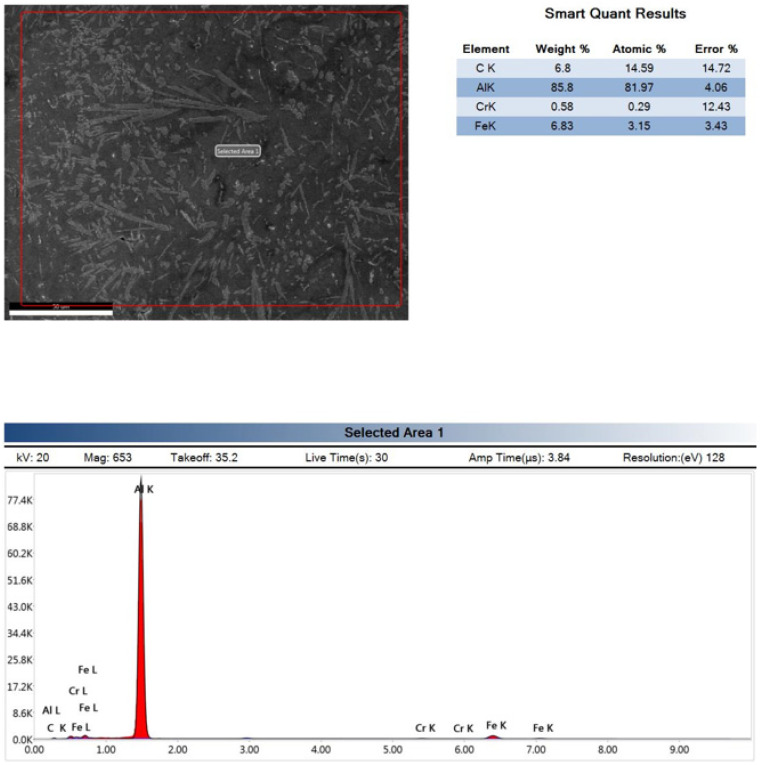
EDX of the Al6061 after 15 min of stirring.

**Figure 10:**
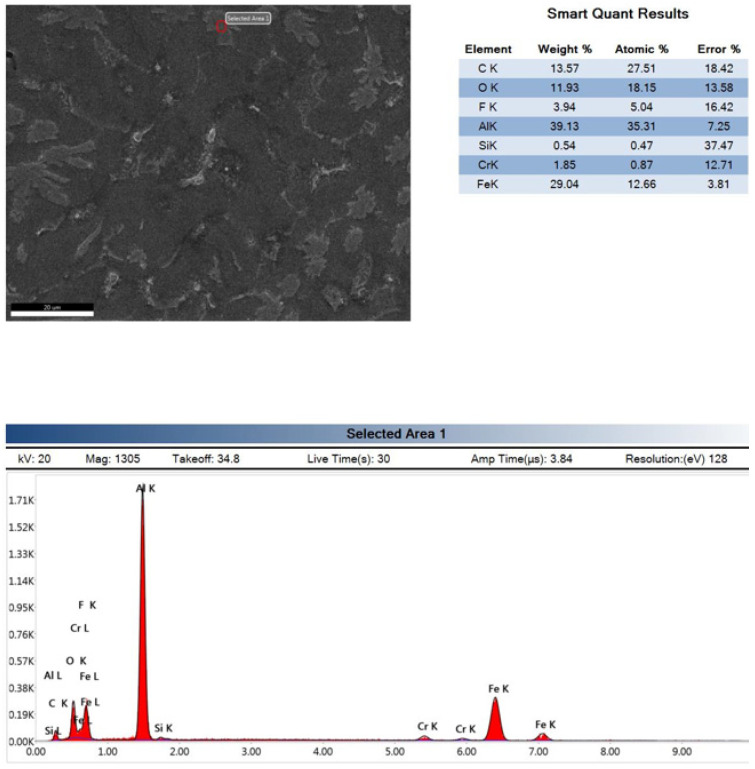
EDX of the Al6061 after 20 min of stirring.

Consequently, EDX testing was conducted for the stirrer material on JEOL; JSM7600F for Al6061 stirred for 15 min and Al6061 stirred for 20 min, as shown in Figure 8, Figure 9 and Figure 10. It is obvious that increasing the stirring time led to the introduction of eroded parts of the stirrer into the Al6061. This analysis illustrates the reasons for the deterioration of the mechanical strength after stirring for long durations.

**Figure 11:**
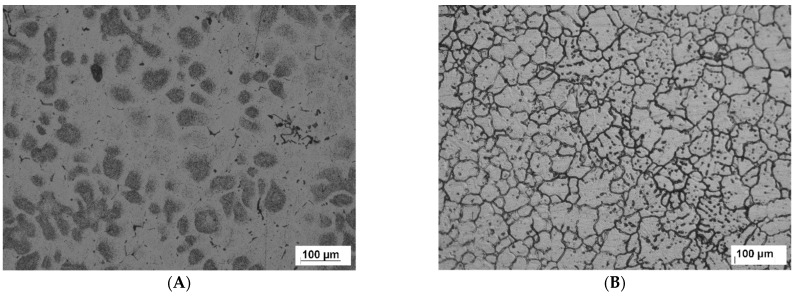

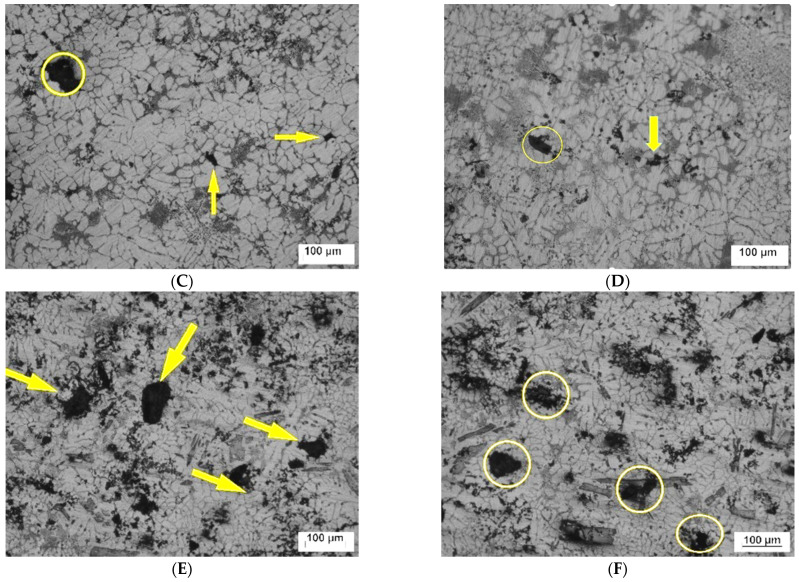
Optical images of Al6061/TiO_2_ micro-composites (**A**) 0%, (**B**) 1 wt.%, (**C**) 2 wt.%, (**D**) 3 wt.%, (**E**) 4 wt.% and (**F**) 5 wt.%, stirred at 1000 rpm for 10 min.

The microstructural of Al6061/TiO_2_ composite samples with different loading fractions fabricated using the selected stir casting parameter, stirring at 1000 rpm for 10 min, is illustrated in Figure 11A–F.

Figure 11A shows the microstructure of unreinforced Al6061 alloy. The microstructure has primary α-Al dendrites and eutectic silicon [27]. A dendritic structure of pure aluminum alloy 6061 with no addition was inserted to serve as a basis for comparison with other reinforced alloys, provide an in-depth understanding of changes at the microstructure level, and find reasons for the mechanical response of the samples. The microstructure of Al6061 containing 1 wt.% of micro-TiO_2_ is shown in Figure 11B. It can be observed that the microstructural transformed from the dendritic structure of unreinforced alloy to grain structure. The reinforcing particulates work as motivating positions of the nuclei initiation of grains over the solidification process [28]. This could be interpreted as an improvement in tensile strength resulting from the incorporation of 1 wt.% of micro-TiO_2_, as shown in Figure 5. Homogeneous tiny porosities (yellow arrows) are distributed over the matrix, where the presence of porosity is due to the gases that dissolved during the stirring of molten metal [29]. Increasing the reinforcing microparticles contributed to the grain refinement of aluminum alloy 6061, as shown in Figure 11C, which presents the grain microstructure of reinforced alloy with 2 wt.% micro-TiO_2_. A higher number of reinforcing particles meant a higher number of potential sites for grain nucleation, leading to more refinement of the formed grains, as can be observed by comparing the grain structures shown in Figure 11B,C of samples reinforced with 2 wt.% and 1% micro-TiO_2_, respectively. The amount of porosity increased with increasing the reinforcing particles [30], as seen in the distinct porosity sites (the yellow arrows and circles) recorded throughout the microstructural investigation. This level of grain refinement with a marginal amount of porosity was recorded for the reinforced alloy with 2 wt.% TiO_2_, which explains the tensile test results shown in Figure 5, according to which the reinforced alloy with 2 wt.% TiO_2_ showed the highest UTS. The reinforced composite with 3 wt.% of TiO_2_ was microstructurally investigated in Figure 11D. The porosity levels that accompanied the addition of 3 wt.% of TiO_2_ were higher than those presented with lower loading fractions, as indicated by the yellow arrows and circles. Increasing the reinforcement content often produces higher porosity amounts because of the nucleation of pores at the TiO_2_ particle surfaces, leading to a decreased flow of liquid metal [31]. This led to a lower strength, compared with low-fraction loadings (Figure 5). The microstructural examination of Al6061/TiO_2_ (4 wt.%) is revealed in Figure 11E. It is clear that the porosity locations increased (marked by yellow arrows and circles) because of the great tendency of the particles to agglomerate, especially at higher percentages of reinforcement leading [32]. The elevated porosity content validated the tensile test results, based on which the increase in the porosity led to a deterioration in the composite UTS, as shown in Figure 5. At 5 wt.% of TiO_2_, shown in Figure 11F, the porosity sites (yellow circles) were the prominent feature of the microstructural examination. The porosity increased with rising the content fraction of reinforcement particulates [33,34,35]. The higher porosity level also reflects a decrease in UTS, shown in Figure 5.

**Figure 12:**
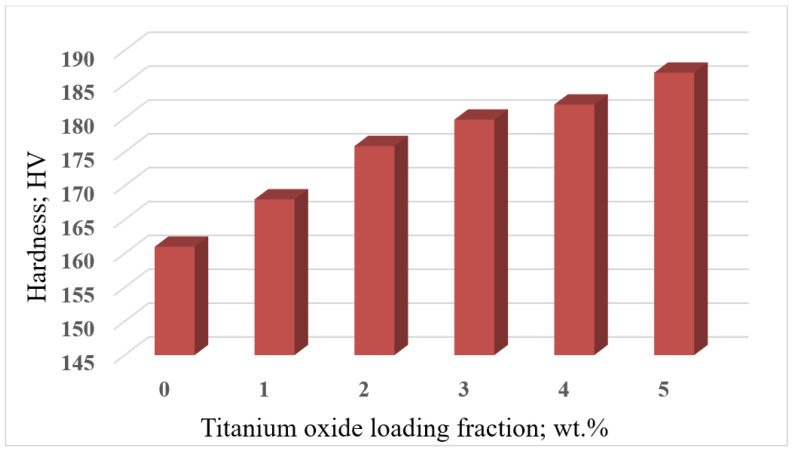
Vickers hardness of Al6061/TiO_2_ with different loading fractions.

The hardness of the Al6061/TiO_2_ composites was measured using a Vickers macro-hardness tester. The average values for the hardness of the synthesized composites are illustrated in Figure 12. The results show that by increasing the loading fraction of the TiO_2_ particles, an increase in the composite hardness occurred. The pure Al6061 recorded a value of 167 VHN, whereas the Al6061/TiO_2_ (5 wt.%) recorded a value of 186.7 VHN. This enhancement in hardness could be attributed to the presence of hard TiO_2_ particulates that impart their hardness to the matrix, as well as to the grain refinement observed over the microstructure resulting from the reinforcement particles. Similar results were concluded by [36,37,38]. Furthermore, the enhancement in the hardness of the composite provides evidence for the good distribution of reinforcement particles inside the matrix [39,40].

## 4. Conclusions

In this study, we experimentally evaluated the evolution of utilizing the stir casting technique on the mechanical properties of Al6061/TiO_2_ composites. Different stirring speeds and duration times were applied to identify the optimum stirring parameters. Utilizing the optimum stirring parameters, the effect of reinforcing Al6061 with different loading fractions was evaluated. The microstructure of the produced composites was also investigated. The following conclusions can be drawn:

The optimum stirring speed is 1000 rpm, with a duration time of 10 min. Increasing the stirring time by more than 10 min leads to an increase in the composite porosity, resulting in a deterioration in the mechanical properties.

Furthermore, the values of 2 wt.% and 5 wt.% of the TiO_2_ recorded the highest ultimate tensile strength and hardness, respectively.

Optical micrographs exhibited a transformation from the dendritic structure of unreinforced alloy into a grain structure with the presence of reinforcing particles.

## Data Availability

The data presented in this study are available on request from the corresponding authors.
